# Live Poultry Exposures, Hong Kong and Hanoi, 2006

**DOI:** 10.3201/eid1307.061031

**Published:** 2007-07

**Authors:** Richard Fielding, Tran H. Bich, La Ngoc Quang, Wendy W.T. Lam, Gabriel M. Leung, Truong Q. Tien, Ella Y.Y. Ho, Le V. Anh

**Affiliations:** * University of Hong Kong, Hong Kong Special Administrative Region, People’s Republic of China; † Hanoi School of Public Health, Ha Noi, Vietnam; ‡ Chinese University of Hong Kong, Shatin, New Territories, Hong Kong Special Administrative Region, People’s Republic of China

**Keywords:** Influenza virus, H5N1 subtype, poultry, population exposures, Hong Kong, Vietnam, risk perception, dispatch

## Abstract

Since 1997, the largest epidemic of highly pathogenic avian influenza (H5N1) ever recorded has caused 172 human and several billion bird deaths. Recently administered questionnaires determined that live poultry exposures have declined by ≈63% in Hong Kong since 2004 and that, in Vietnam, domestic backyard exposures to poultry are likely more important than retail exposures.

Most human cases of highly pathogenic avian influenza H5N1 (HPAI) arise from exposure to infected poultry (*1**–**3*; Figure). Mapping poultry exposure and its determinants can enhance HPAI surveillance (*4*). We compared live poultry exposures in both Hong Kong Special Administrative Region and Vietnam in 2006 and examined changes in levels of exposure in Hong Kong since 2004, when a similar survey was performed in Hong Kong (*4*).

**Figure:**
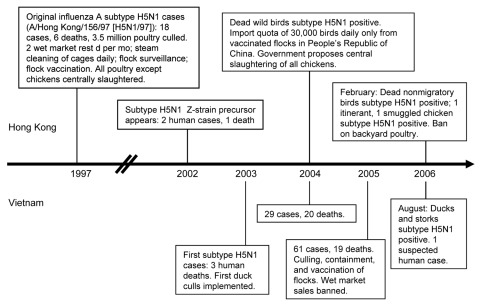
Chronology of influenza A (H5N1) outbreaks and responses, Hong Kong and Vietnam. Double slashes represent a break in the timeline.

## The Study

In Hong Kong, random household telephone interviewing of 1 adult >17 years of age selected by Kisch grid (which randomizes selection of persons within households) was conducted from December 2005 through 2006 from a list of 5,000 numbers. Simultaneously in Vietnam, stratified cluster sampling was carried out throughout 2 districts in each of 5 northern provinces. Within 3 of these provinces, 1 district with and 1 without an HPAI epidemic history were selected. Within each district, 1 urban and 1 rural commune each provided 100 households randomly selected from electoral rolls. Kisch grid selected 1 adult from each household for face-to-face interviews.

Respondents estimated their live poultry purchase frequency and touching at purchase (*4*). We attributed standard values to respondents’ reports (e.g., monthly = 12; weekly = 52) (*4*) to give standardized household purchases of live poultry. Multiplying standard purchases by reported buying frequencies standardized buying patterns (*4*). Self-reported buyer touching of birds during purchase was standardized by adjustment for reporting differences by gender proportion weighting (*4*) and reported touching frequency to calculate adjusted buying exposures. Vietnam also surveyed backyard poultry practices. Households raising poultry reported the number, type, changes in husbandry practices, and poultry deaths for the past 12 months.

## Conclusions

In Hong Kong, 2,784 contacts yielded 1,760 interviews (return rate 63%); 64% of respondents were women and 36% were men; their median age, 44 years (Table 1). Vietnam’s census-derived sample frame comprised 2,412,000 of 18,264,000 national households; 1,988 (0.01% of all Vietnamese households) formed the sample. Of 1,196 (60%) female and 792 (40%) male participants, the median age was 39 years, 50% lived in urban and 50% in rural communes, and >11% had primary education only (Table 1).

**Table 1:** Sample characteristics and population censuses, Hong Kong and Vietnamese samples

	Survey Vietnam; Hong Kong, %	Census Vietnam*; Hong Kong,† %	Effect size‡ Vietnam; Hong Kong
Sex			
Male	39.8; 35.9	46.1; 47.8	0.19; 0.24
Female	60.2; 64.1	50.9; 52.2	
Age, y			
15–24	14.2; 10.5	28.1; 11.1	0.50; 0.21
25–34	22.5; 14.4	21.7; 18.1	
35–44	25.3; 27.0	20.4; 22.7	
45–54	22.4; 27.0	13.7; 21.2	
55–64	13.2; 10	6.9; 11.9	
>65	2; 11	9.3; 14.9	
Residence			0.54
Urban	50	26.3	
Rural	50	73.7	
Education			0.32
None or kindergarten	3.9	8.4	
Primary	14.7	20.5	
Secondary	49.5	45.2	
Matriculation	6.3	9.4	
Tertiary/above	25.3	16.4	

*Reference (*5*). †Reference on education (*6*). On sex and age, mid-2006, available from www.censtatd.gov.hk/hong_kong_statistics/statistical_tables/index.jsp ‡Three levels of effect size: 0.1 small, 0.3 medium, 0.5 large.

In Hong Kong in 2006, 18,586 standardized purchases averaged 10.56 chickens/household/year (for men, 9.4, for women, 11.2). This is a territory-wide gender-adjusted rate of 11.05 chickens/household/year, which indicated that 22,673,000 live chickens were purchased during the preceding year, 41% fewer than in 2004. Households buying poultry bought an average of 15.6 chickens/household/year. Among respondents personally buying, 7.5% touched the poultry during purchasing (compared with 11% in 2004), giving ≈1,700,500 exposures/year. Adjustment for touching frequency and gender differences in reported touching (males 6.7%, females 4.7%) gave ≈1,110,900 contacts (4.9%, 95% confidence limit [CL] 3.8–6) for Hong Kong in 2006, or 0.76 exposures/buying household/year (0.23/person/year, if one assumes 3.36 persons/household). Applied retrospectively to Hong Kong 2004 data, this gave an adjusted exposure rate of 8.6% (95% CL 6.8–10.3), ≈3,311,300 contacts, and exposure frequency of 2.07 exposures/buying household/year (0.62/person/year) in 2004. These adjusted estimates indicate an absolute exposure decline of 3.7% (95% CL 2.25%–4.91%), a relative decline of 43% between 2004 and 2006. Less purchasing and touching reduced annualized buying exposures by 63% overall.

In Vietnam, respondents reported 10,659 standardized purchases, averaging 5.36 chickens/household/year, giving a gender-adjusted (male 5.5, female 5.3) rate of 5.43 chickens/household/year. Estimated number of live birds purchased in the sampled provinces (5.43 × 2,412,000 households) was 13,097,000 chickens per year. Buying households (820,080, 34%) buy on average 15.97 chickens per year, comparable to the Hong Kong 2006 purchase rate. Touching frequency during purchasing (overall 68%, 64%–71%; women 70% [67%–73%], men 54% [51%–57%]; χ^2^ = 45.57, df = 4, p<0.001), after adjustment for gender proportion and reported touching, was 63% (62%–64%). Estimated exposures in the surveyed provinces from buying were ≈13,097,000 × 0.63 = ≈8,251,000 exposures/year. When these rates were used, national per capita exposure estimates (assuming 4.49 persons/household) from touching when buying are ≈62,479,000 exposures/year, 2.24 exposures/person/year in buying households, 0.76 exposures/person/year overall.

In the 1,150/1,988 households (58%) that raised poultry, 92 (5%) ceased keeping poultry from February 2005 through February 2006 (Table 2). Households kept a median of 9 chickens. Overall, 22% of those keeping backyard poultry reportedly had birds die in the previous year. Of these, 12% of households threw the dead bird away without informing authorities, 9% informed the authorities, and 5% sold or ate the dead bird. Of those reporting bird deaths, 214 (84%) had been ordered by officials to destroy some or all of their birds. Incidence of bird deaths was greater in rural areas (52% vs. 48%, Fisher p<0.001), but rural residents threw them away (68% vs. 32%, Fisher p = 0.031) or sold or ate them (87% vs. 13%, Fisher p = 0.006) more often than did urban residents.

**Table 2:** Numbers of households rearing domestic poultry, Vietnam, 2006 (proportions)

Poultry	No. households			
No. birds raised	1–5	6–10	11–20	>21 (range)
Type				
Fighting cocks	40	5	1	2 (21–50)
Chickens	284	283	257	155 (21–800)
Ducks	52	14	5	14 (21–500)
Geese/swans	42	22	11	8 (21–70)
Ornamental	32	10	4	1 (21–30)

While 34% (32%–36%) of households buy live chickens, 53% (52%–54%) (1,278,360) raise live poultry at home, and 12% (10%–13%) do both. Assume a 53% national average and, conservatively, that all persons within households rearing backyard poultry have at least weekly physical contact with their birds, bird eggs, or feces. Household size in the surveyed districts averages 3.38 persons (General Statistics Office, Hanoi). Thus, 224,685,500 exposures/year would occur from backyard poultry in surveyed districts, an average exposure within backyard poultry raising households of ≈175 exposures/person/year. Households buying live poultry have 8,251,000 /820,080 = 10.1 exposures/household/year (2.99 exposures/person/year) from these purchases. Total purchase-related plus backyard exposure events then equal (10.1 × 820,080) + 224,685,500 = 232,968,300 exposures/year. Average household exposure is therefore ≈96 exposures/household/year (28 exposures/person/year) in sampled districts. If daily backyard exposure occurs, then there are ≈1,581,081,300 total exposures, ≈655 exposures/household/year (194 exposures/person/year). Nationally, average household size is 4.49 persons. Hence, between ≈2,322,546,000 (weekly contact) and ≈15,882,953,000 (daily contact) exposures/year, ≈127–869 exposures/household/year occur nationally. If multiple contacts occur daily, these figures would be much higher.

Epidemic and nonepidemic district-buying frequency CL overlapped (exposure 3.4 [1.9–4.8] chickens/household/year vs. nonexposure 5.8 [4.5–7.0] chickens/household/year). Dual adjusted touching frequencies were 69% (62%–76%) in epidemic and 60% (57%–63%) in nonepidemic districts, respectively. Backyard poultry were more common in epidemic districts (71% [67%–75%] vs. 45% [42%–48%]), where keeping poultry declined 17% (14%–20%) compared with 8% (6%–10%) in nonepidemic districts. Epidemic and nonepidemic districts had comparable average incomes (t = 0.832, df 1,283.9, p = 0.406).

In Hong Kong, government import restrictions have reduced poultry availability by 41% from 2004 to 2006. Purchase and touching declines prompted by health education messages have together reduced exposure by ≈60%.

Fewer Vietnamese households bought live chickens, but those that did so bought at comparable frequencies to Hong Kong 2006 households. Chickens are relatively more expensive in Vietnam. Adjusted for purchasing power parity (www.worldbank.org/data/quickreference/quickref.html), live chickens costs $16.6–$18.0 and $21.8–$31.0 (international dollars; www.worldbank.org/data/quickreference/quickref.html) each in Hong Kong and in Vietnam, respectively. Hence, temptation to use sick, dying, or dead poultry is high, increasing the risk for human influenza (H5N1) infection (*7*). Average Vietnamese exposure range from backyard sources (28–194 exposures/person/year) is 100–700× higher than Hong Kong 2006 exposures from purchasing (0.23 exposures/household/year). If 53% of Vietnamese households average 9 birds each and if 22% of these households (2,129,582) had only 1 bird die, a 5% consumption rate of the dead birds means that 106,500 sick or dying birds are consumed annually, posing a major health threat (*7*). This is a risk that governments must urgently target.

Limitations include generalizing from 5 northern Vietnamese provinces to the country as a whole and using arbitrary estimates for backyard exposure frequency. Nonetheless, valuable data are presented on differential exposure patterns.
